# Low-grade oligodendroglioma of the pineal gland: a case report and review of the literature

**DOI:** 10.1186/1746-1596-5-59

**Published:** 2010-09-17

**Authors:** Georgia Levidou, Penelope Korkolopoulou, George Agrogiannis, Nikolaos Paidakakos, Dimos Bouramas, Efstratios Patsouris

**Affiliations:** 1 National and Kapodistrian University of Athens, Department of Pathology, Athens, 11527, Greece; 2 Department of Neurosurgery, Athens Naval Hospital, Athens 11521, Greece

## Abstract

**Background:**

Gliomas are a very rare subtype of pineal region tumours, whereas oligodendrogliomas of the pineal region are exceedingly rare, since there have been only 3 cases of anaplastic oligodedrogliomas reported this far.

**Methods-Results:**

We present a case of a low-grade oligodendroglioma arising in the pineal gland of a 37 year-old woman. The patient presented with diplopia associated with a cystic pineal region mass demonstrated on MRI. Total resection was performed and histological examination showed that the cystic wall consisted of tumour cells with a central nucleus a perinuclear halo and minimal pleomorphism. Immnunohistochemical analysis showed that these cells were diffusely positive for CD57, and negative for GFAP, CD10, CD99, cytokeratins, neurofilaments and synaptophysin. FISH analysis was performed in a small number of neoplastic cells, which were not exhausted after immunohistochemistry and did not reveal deletion of 1p and 19q chromosome arms. However, the diagnosis of a low grade oligodendroglioma of the pineal gland was assigned.

**Conclusion:**

Although the spectrum of tumours arising in the pineal gland is broad, the reports of oligodendrogliomas confined to this location are exceedingly rare, and to the best of our knowledge there is no report of a low-grade oligodendroglioma. However, they should be added in the long list of tumours arising in the pineal gland.

## Introduction

Neoplasms of the pineal gland are comparatively rare, comprising only 0.4 to 1% of all intracranial tumours [1,2]. Despite their low incidence, they encompass a broad spectrum of distinct histological tumour types. This diversity probably reflects the wide range of the normal cell types that reside in the pineal gland and its adjacent structures. Tumours of the pineal gland are further classified as the pineal parenchymal cell derivatives (pineal parenchymal tumours, PPTs), which account for 22.4% to 42% of all neoplasms arising in this region, [3-5], and those of germ cell origin [3]. The other tumour types less frequently encountered in the pineal region are meningioma, choroid plexus papilloma and craniopharyngioma [3].

Gliomas are a very rare subtype of pineal region tumours, which are thought to arise from the surrounding glial stroma [6]. The majority of the published cases belongs to the group of astrocytic tumours and includes mainly pilocytic astrocytoma, anaplastic astrocytoma and glioblastoma [7,8], low-grade/diffuse astrocytomas being extremely rare [8-10]. Oligodendrogliomas of the pineal region are exceedingly rare. To the best of our knowledge there have been only 3 cases of anaplastic oligodedrogliomas reported this far [3,11], whereas there have been no reports of low-grade tumours. We describe a low-grade oligodendroglioma appearing as a cystic lesion restricted to the pineal body and review the relevant literature.

## Case Report

**Figure 1:**
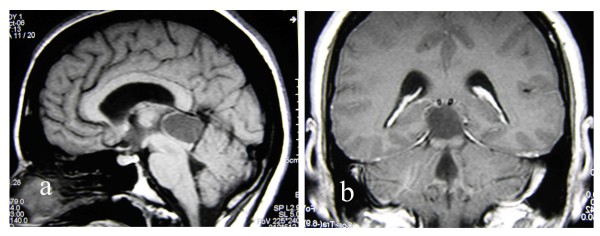
**Pre-operative MR images illustrating the presence of a cystic lesion located in the pineal region**.

A 37 year old woman presented to the Athens Naval Hospital with a slight impairment of gaze. She denied having any other focal symptoms, such as nausea and vomiting or any previous relevant history. Neurological examination failed to reveal any other signs apart from upward diplopia. All laboratory findings were within normal limits. She underwent an MRI of the brain, which disclosed a cystic pineal/posterior third ventricular region mass. Written informed consent was obtained from the patient for publication of this case report and accompanying images. A copy of the written consent is available for review by the Editor-in-Chief of this journalOn radiological grounds, the tumour mass seemed to be sharply demarcated and had a homogenous peripheral enhancement. (Figure 1).

### Operation

The patient underwent a total excision of this cystic lesion, through an midline infratentorial supracerebellar approach to the pineal region. After a slight transposition of the Galenic venous system a cystic mass with a yellowish content was revealed, that was removed by gradually detaching the wall of the cyst from the surrounding neurovascular structures, down to the compressed tectal plate. Intra-operative inspection revealed a well demarcated cystic lesion, without showing any infiltration of adjacent structures. There was slight haemorrhage in this area, which was controlled with haemostatic agents.

### Histological findings

The resected lesion was fixed in formalin solution and routinely processed in paraffin for light microscopy and immunohistochemistry.

**Figure 2:**
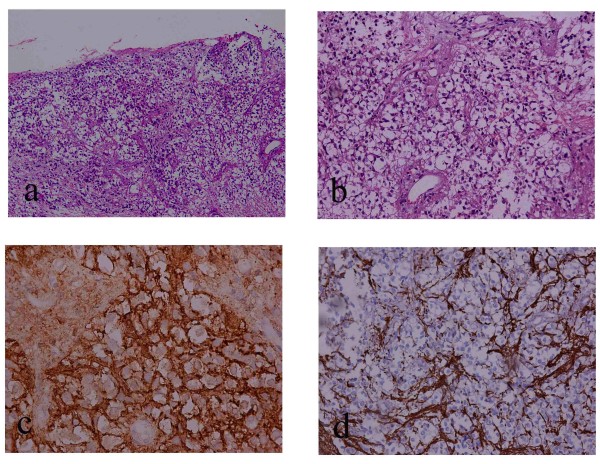
**a. Section showing the presence of neoplastic cells with swollen clear cytoplasm within the cystic wall (MX100)**. b. The neoplastic cells had a centrally located round nucleus with open chromatin, a perinuclear halo and did not display any pleiomorphism (MX400). c. d. The tumour cells were positive for CD57 and negative for GFAP staining.

Microscopic examination of hematoxylin and eosin stained sections showed that the cystic lesion wall was composed of white matter partially covered by cuboidal cells. Within the cyst wall there were aggregates of uniformly round neoplastic cells with characteristic perinuclear haloes and a centrally located round nucleus with open chromatin and without any pleomorphism (Figure 2a, b). Between these cells there was a dense network of branching capillaries. Moreover, detailed examination did not reveal the presence of any Rosenthal fibers, granular bodies or necrosis. Immunohistochemical analysis showed that the neoplastic cells were positive for CD57 and negative for EMA, Ker 18, Ker 7, synaptophysin, neurofilaments, CD99 and CD10, as well as glial fibrillary acidic protein (GFAP) (Figure 2c, d). The Ki67 proliferation index was very low (approximately 1% of neoplastic cells). We tried to perform FISH analysis for chromosome arms 1p and 19q. Given that the neoplastic tissue was almost exhausted during immunohistochemistry, we were able to analyze only a small number of neoplastic cells, which did not show 1p and 19q deletion. Therefore, on the basis only of morphology and immunophenotype a diagnosis of oligodendroglioma, Grade II was assigned.

### Postoperative course

Post-operativeMRI performed one day after surgery did not demonstrate the presence of any residual pathologic tissue. The patient was also examined monthly after the surgery and was always free of any symptoms. In the 6 months follow-up in MRI there was still no evidence of recurrence.

## Discussion

Oligodendrogliomas are the third most common tumour of glial origin and may be diagnosed at any age with two peak incidences: 6 to 12 years and 26 to 46 years [12,13]. They have a predilection for the cortex and the subcortical white matter, yet it is suggested that they may develop in any location throughout the neuraxis [12,13]. Thus, there are reports of patients with cerebellar, spinal cord and primary leptomeningeal oligondedrogliomas [14].

The pineal gland (weighting approximately 0.1 g) is located at the posterior aspect of the third ventricle and the anterior aspect of the splenium of the corpus callosum and tela choroidea [15]. The principal cell is the pineal parenchymal cell or pineocyte, which is surrounded by a stroma of fibrillary astrocytes admixed with sympathetic neurons [16-18]. The presence of glial cells in the pineal gland was first demonstrated by impregnation techniques with gold chloride-sublimate [15,19]. Later Papasozomenos et al established the presence of glial fibrillary acidic (GFA) protein-containing cells in the human pineal gland, suggesting the significance of astrocytic participation in the structure of human pineal gland throughout life [6,10]. Moreover, according to Burger et al [5], definite proof of the incospicious existence of pineal astrocytes comes from the occurrence of gliotic tissue in lesions such as craniopharyngiomas and hemangioblastomas. In addition to fibrillar astrocytes, there are some oligodendrocytes which are restricted to the proximal part of the stalk, near the deep pineal tissue where myelinated axons are abundant [18,20]. Adjacent to the gland there are ependymal cells and cells forming the choroid plexus of the third ventricle, as well as glial cells from the brainstem [21]. Most tumours arising in the pineal region reportedly result from malignant transformation of the pineal parenchymal cells, the surrounding stromal or so called "interstitial" cells or the cells comprising the adjacent tissues [3], a fact which along with the presence of fibrillary astrocytes and oligodendrocytes in the pineal parenchyma, supports the concept that gliomas restricted in the pineal region presumably originate from pineal glial cells. However, the few gliomas reported in the literature usually involved the adjacent brain tissues as well, so that it is not very clear whether the resected tumour arose primarily from the pineal gland or involved it secondarily [3,7-9,22]. In our case the conclusion that the tumour arose from the pineal gland was based on postoperative MRI, intraoperative inspection, and the excellent postoperative course of the patient after complete excision of the epiphesial lesion.

Gliomas of the pineal region are reported to be of two varieties, distinguishable by the presence or absence of a cyst [8]. The cystic variety has been quoted to have more benign characteristics and clinical course [8] and tends to be restricted in the pineal gland. The patient reported by Das et al. [11], suffered from a cystic anaplastic oligodendroglioma in the pineal region and had excellent clinical course after complete tumour resection, despite the quoted rather poor prognosis of anaplastic oligodendrogliomas in other locations. Difficulty in diagnosis most frequently arises when trying to distinguish low-grade gliomas in the pineal region from the more common pineal cyst. Both lesions typically present with hydrocephalus or Paurinaud sundrome due to tectal compression [8]. Imaging studies can be useful, but as in our case cannot reliably lead to a definite diagnosis. Pineal cysts may be as large as 4.5 cm and often have a peripheral calcification, whereas cystic gliomas are more likely to have an enhancing soft tissue component [15]. However, most of the lesions display overlapping findings and therefore histological examination for the differential diagnosis is essential. In sections, pineal cysts are typically composed of a thin gliotic wall with scattered Rosenthal fibers and have well defined borders from the adjacent parenchyma [15]. The presence of tumour cells with a characteristic perinuclear clearing on paraffin section should raise the possibility of the presence of an oligodendroglial neoplasm. Though both pineal cysts and cystic low grade oligodendrogliomas have a benign clinical course, their differential diagnosis is significant since the latter have low potential for recurrence and should be followed accordingly.

Moreover, clear cell ependymomas, pineocytomas and metastatic carcinomas of the pineal region must be considered in the differential diagnosis of low-grade oligodendrogliomas of the pineal gland. Ependymomas of the pineal region are quite infrequent [23], whereas there are few reports of a solitary carcinoma metastasis in this location [24-26]. The presence of ependymal and perivascular rosettes, immunoreactivity for GFAP (although oligodenrogliomas may also by positive for GFAP) and absence of CD57 and CD99 expression can be helpful for the establishment of a diagnosis of ependymoma [27]. Furthermore, immunopositivity of cytokeratins and absence of GFAP expression are useful markers for the distinction of a metastatic carcinoma located in the pineal region from a low grade oligodendroglioma. Pineocytomas should be considered of course, in the differential diagnosis of any lesion located in the pineal gland. However, in our case the diagnosis of pineocytoma was excluded on the basis of the morphological features of the neoplastic cells, along with the absence of neurofilaments expression [14].

However, the histological recognition of oligodendroglial tumours is still subject to a significant observer bias since the morphologic criteria are vague and subjective [28,29]. New oligodenroglioma markers, such as OLIG1/2 and CD57 have invariably improved the accuracy of the diagnosis of oligodenroglial tumours [28,29]. The same applies to genetic markers, such as loss of heterozygosity (LOH) in 1p and 19q chromosome [28,29]. The application of these techniques in routine diagnosis could be one of the reasons accounting for the relative increase in the incidence of oligodendroglial tumours in uncommon sites, such as the pineal gland. In our case, due to the relative small amount of neoplastic tissue which was almost exhausted during immunohistochemistry, FISH analysis for the detection of these 19q/1p LOH was performed in a small number of neoplastic cells and was negative. The small number of these remaining cells possibly reduces the reliability of the results of the molecular analysis. Moreover, it is noteworthy that there exists a significant percentage of low-grade oligodendrogliomas that do not show 1p and 19q LOH, denoting that the diagnosis of oligodendrogliomas cannot be established only on the presence of these molecular alterations [30,31].

In conclusion, despite the very low incidence of pineal gland oligodendrogliomas, neurosurgeons and neuropathologists should be alert for their presence because despite their excellent course, they still carry the potential for evolution and recurrence as well as spread and metastasis.

## Competing interests

The authors declare that they have no competing interests.

## Authors' contributions

GL: Editing the manuscript and diagnosing the case, PK: Editting the manuscript and diagnosing the case, GA: Diagnosing the case, NP: Managing and operating the patient, Editting the manuscript, DB: Managing and operating the patient, EP: Supervising the whole attempt from the diagnosis of the patient to the submission of the manuscript. All authors read and approved the final manuscript.
